# Ten-year persistence and evolution of *Plasmodium falciparum* antifolate and anti-sulfonamide resistance markers *pfdhfr* and *pfdhps* in three Asian countries

**DOI:** 10.1371/journal.pone.0278928

**Published:** 2022-12-16

**Authors:** Suttipat Srisutham, Wanassanan Madmanee, Jindarat Kouhathong, Kreepol Sutawong, Rupam Tripura, Thomas J. Peto, Rob W. van der Pluijm, James J. Callery, Lek Dysoley, Mayfong Mayxay, Paul N. Newton, Tiengkham Pongvongsa, Bouasy Hongvanthong, Nicholas P. J. Day, Nicholas J. White, Arjen M. Dondorp, Mallika Imwong

**Affiliations:** 1 Faculty of Allied Health Sciences, Department of Clinical Microscopy, Chulalongkorn University, Bangkok, Thailand; 2 Faculty of Tropical Medicine, Mahidol-Oxford Tropical Medicine Research Unit, Mahidol University, Bangkok, Thailand; 3 Buntharik Hospital, Amphoe Buntharik, Ubon Ratchathani, Thailand; 4 Nuffield Department of Medicine, Centre for Tropical Medicine and Global Health, University of Oxford, Oxford, United Kingdom; 5 Center for Parasitology Entomology and Malaria Control (CNM), Phnom Penh, Cambodia; 6 Institute of Research and Education Development, University of Health Sciences, Ministry of Health, Vientiane, Lao PDR; 7 Lao-Oxford-Mahosot Hospital-Wellcome Trust Research Unit, Microbiology Laboratory, Mahosot Hospital, Vientiane, Lao PDR; 8 Savannakhet Provincial Health Department, Phonsavangnuea Village, Kaysone-Phomvihan District, Savannakhet, Laos; 9 Center of Malariology, Parasitology and Entomology, Ministry of Health, Vientiane, Laos; 10 Department of Molecular Tropical Medicine and Genetics, Faculty of Tropical Medicine, Mahidol University, Bangkok, Thailand; Johns Hopkins University Bloomberg School of Public Health, UNITED STATES

## Abstract

**Background:**

The amplification of *GTP cyclohydrolase 1* (*pfgch1*) in *Plasmodium falciparum* has been linked to the upregulation of the *pfdhfr* and *pfdhps* genes associated with resistance to the antimalarial drug sulfadoxine-pyrimethamine. During the 1990s and 2000s, sulfadoxine-pyrimethamine was withdrawn from use as first-line treatment in southeast Asia due to clinical drug resistance. This study assessed the temporal and geographic changes in the prevalence of *pfdhfr* and *pfdhps* gene mutations and *pfgch1* amplification a decade after sulfadoxine-pyrimethamine had no longer been widely used.

**Methods:**

A total of 536 *P*. *falciparum* isolates collected from clinical trials in Thailand, Cambodia, and Lao PDR between 2008 and 2018 were assayed. Single nucleotide polymorphisms of the *pfdhfr* and *pfdhps* genes were analyzed using nested PCR and Sanger sequencing. Gene copy number variations of *pfgch*1 were investigated using real-time polymerase chain reaction assay.

**Results:**

Sequences of the *pfdhfr* and *pfdhps* genes were obtained from 96% (517/536) and 91% (486/536) of the samples, respectively. There were 59 distinct haplotypes, including single to octuple mutations. The two major haplotypes observed included **IRN**I-**AGE**AA (25%) and **IRNL**-S**G**K**G**A (19%). The sextuple mutation **IRNL**-S**G**K**G**A increased markedly over time in several study sites, including Pailin, Preah Vihear, Ratanakiri, and Ubon Ratchathani, whereas **IRNI**-**AGE**AA decreased over time in Preah Vihear, Champasak, and Ubon Ratchathani. Octuple mutations were first observed in west Cambodia in 2011 and subsequently in northeast Cambodia, as well as in southern Laos by 2018. Amplification of the *pfgch1* gene increased over time across the region, particularly in northeast Thailand close to the border with Laos and Cambodia.

**Conclusion:**

Despite the fact that SP therapy was discontinued in Thailand, Cambodia, and Laos decades ago, parasites retained the *pfdhfr* and *pfdhps* mutations. Numerous haplotypes were found to be prevalent among the parasites. Frequent monitoring of *pfdhfr* and *pfdhps* in these areas is required due to the relatively rapid evolution of mutation patterns.

## Introduction

Malaria remains a serious public health concern worldwide, with 229 million cases reported in 2019 [1] *Plasmodium falciparum* has been responsible for a substantial number of severe clinical cases and deaths. The spread of drug-resistant forms of *P*. *falciparum* has posed a serious danger to malaria control efforts. In Thailand, sulfadoxine-pyrimethamine (SP) replaced chloroquine (CQ) as the first-line treatment in 1973 due to widespread *P*. *falciparum* chloroquine resistance. Moreover, the significant decline in the efficacy of SP in Thailand necessitated a shift in the first-line treatment in 1991 [2]. In Cambodia, chloroquine (CQ) was used as the first-line treatment in the middle of the 20^th^ century and resistance to CQ was first reported in the early 1960s [3]. Sulfadoxine-pyrimethamine (SP) then replaced CQ and was used for approximately ten years until resistance to SP emerged in the late 1970s [4]. Chloroquine and sulfadoxine-pyrimethamine can no longer be used in Cambodia due to persistence of molecular markers of resistance to these drugs [5]. Mefloquine was subsequently introduced as a first-line treatment in the 1980s [6]. Mefloquine continues to be used as the partner drug in the artemisinin combination therapy (ACT) artesunate-mefloquine (AM), which was adopted as first-line treatment across most of the country around 2016. In Lao People’s Democratic Republic (PDR), SP was a second-line drug but was rarely used until 2005. Artemether-lumefantrine (AL) is currently the first line of treatment [7].

SP serves as a synergistic inhibitor of folate in *P*. *falciparum* by targeting the enzymes dihydropteroate synthase (DHPS) and dihydrofolate reductase (DHFR) [8]. *In vitro* and in vivo investigations have shown that SP resistance is mostly caused by amino acid point mutations at codons N51I, C59R, S108N, and I164L of PfDHFR and S436A, A437G, K540E, A581G, and A613S of PfDHPS [9]. *Pfdhfr* (dihydrofolate reductase) and *pfdhps* (dihydropteroate synthase) gene mutations have been commonly employed as genetic indicators for SP resistance surveillance [10, 11]. However, evidence has shown that the Asn mutation in DHFR codon 108 was rather common [12]. Antifolate resistance has also been linked to the amplification of the gene for *P*. *falciparum guanosine triphosphate cyclohydrolase 1* (*pfgch1*), which codes the key enzyme, Guanosine triphosphate (GTP) cyclohydrolase I (GCH1). The enzyme catalyzes the conversion of GTP into dihydroneopterin triphosphate (DHNP) in the folate pathway. An increased copy number of the malaria parasite *P*. *falciparum* GCH1 gene has been reported to influence antimalarial antifolate drug resistance evolution [13]. Although SP had been withdrawn in Thailand for many years, antifolate and anti-sulfonamide resistance markers in falciparum malaria continue to be prevalent in Thailand’s border regions [14]. As such, studies on the current status of antifolate and anti-sulfonamide resistance markers in *P*. *falciparum* in Cambodia and Lao PDR are needed given that several factors, such as drug target, nature of genes, and host/parasite genetic background, may affect the persistence of SP resistance differently after SP use is discontinued.

The current study aimed to determine the frequencies of *pfdhfr* and *pfdhps* gene mutations and haplotypes in *P*. *falciparum* isolates from Thailand, Cambodia, and the Lao PDR, as well as the prevalence of *pfgch1* gene copy number variations, in order to determine the current status of the SP resistance markers.

## Methods

### Specimen collection

Specimens were collected from Thailand (n = 144) in 2014 and 2016–2018; Cambodia (n = 286) in 2008, 2011, and 2016–2018; and Lao PDR (n = 106) in 2010, 2011, 2013–2014, and 2015. Our study protocol was approved by the Ethical Review Committee of the Faculty of Tropical Medicine, Mahidol University (Bangkok, Thailand) (approval no. MUTM 2012-045-05).

### DNA extraction and PCR

Genomic DNA was extracted from samples collected from patients with confirmed *P*. *falciparum* infection. The *pfdhfr* and *pfdhps* genes were amplified through nested PCR, with the conditions for amplification having been previously described [15–18]. The mutations in the *pfdhfr* and *pfdhps* genes were subsequently detected by Sanger sequencing (Macrogen, Korea). The *pfgch1* gene amplification was analyzed using real-time PCR based on a previously described protocol [19].

### Statistical analysis

The *pfdhfr*, *pfdhps*, and *pfgch1* gene copy numbers were described as the proportion of each haplotype presented at each study site for each year. The changing trend in molecular markers was analyzed by comparing the prevalence of markers over time according to year. Genetic diversity within populations was estimated by computing haplotype diversity (H) diversity [20]. Pearson’s chi-square test was used to compare proportions of haplotypes using SPSS version 28.0 (IBM), with a p-value of 0.05 being considered statistically significant. The dominant amino acid haplotypes were network calculated using the median joining method using Network Software version 10.

## Results

### Prevalence of individual point mutations in *pfdhfr* and *pfdhps*

Sequences of the *pfdhfr* gene were obtained from 96.46% (517/536) of the samples. In Ubon Ratchathani, Thailand, a high prevalence of N51I, C59R, and S108N was observed from 2014 to 2018. a significant increase in I164L mutations was found from 2014 (24/101) to 2018 (8/8) (p < 0.05; S1 Table). In Pailin, Preah Vihear, Pursat, Ratanakiri, and Stung Treng, Cambodia, a high prevalence of N51I, C59R, and S108N mutants was observed over time. In Pailin, Preah Vihear, and Ratanakiri, Cambodia, a significant increase in I164L mutation was identified (p < 0.05). In Champasak, Lao PDR, a high prevalence of N51I, C59R, and S108N mutant alleles was found. A significantly increased prevalence of I164L mutation (p < 0.05) was noted over time in Champasak.

Sequences of the *pfdhps* gene were obtained from 90.67% (486/536) of the samples. In Ubon Ratchathani, Thailand, a high prevalence of S436A, A437G, and K540E was observed in 2014 and 2016–2017. Moreover, a significantly increased amount of A581G was noted from 8% in 2014 to 100% in 2018 (S1 Table). No mutations in A613S were identified. A reduction in S436A and A437G was found. In Cambodia, a high prevalence of A437G mutant was noted over time. Moreover, a significant reduction in the K540E mutation was observed in Pailin and Preah Vihear. No A613S mutations were found in Pailin, Preah Vihear, Pursat, and Ratanakiri. In 2018, A613S mutations were recently found in Stung Treng, near Attapeu and Champasak, Lao PDR, where a high prevalence of A613S mutations had been found. Champasak, Lao PDR had a high prevalence of A613S mutations in 2015 (63%).

### *Pfdhfr* and *pfdhps* haplotypes

In this study, several *pfdhfr* haplotypes were reported, including wild type, single, double, triple, and quadruple mutations including NC**N**I, N**RN**I, **I**C**N**I, **IRN**I, N**RNL**, and **IRNL**. In Ubon Ratchathani, Thailand, an increase in *pfdhfr* quadruple mutant alleles was observed from 24% in 2014 to 100% in 2018 (S1 Fig), whereas a reduction in *pfdhfr* triple mutant alleles was observed from 76% in 2014 to 0% in 2018. In Cambodia, an increase in *pfdhfr* quadruple mutant alleles was found in Pailin and Preah Vihear. In Lao PDR, a high prevalence of triple mutations was observed in Attapeu, Champasak, and Salavan. Quadruple mutations were increased in Champasak.

The current study found several *pfdhps* haplotypes, including wild type, single, double, triple, and quadruple mutations. An increase in double mutations but a reduction in triple mutations were observed in Ubon Ratchathani, Pailin, and Preah Vihear (S1 Fig). In Champasak, an increase in quadruple mutant alleles was observed.

The *pfdhfr* and *pfdhps* haplotype diversity within populations was estimated [20]. The haplotype diversity of the *pfdhfr* gene was increased in Preah Vihear, Pursat, Ratanakiri, Champasak, and Ubon Ratchathani (Table 1). In 2017, a high haplotype diversity of *pfdhfr* and *pfdhps* genes was observed in Champasak.

**Table 1:** Haplotype diversity of *pfdhfr* and *pfdhps* in Thailand, Cambodia, and Lao PDR.

Study site		Study year	Haplotype diversity	
			*pfdhfr*	*pfdhps*
Cambodia	Pailin	2008	0.498	0.462
		2017	0.280	0.249
	Preah Vihear	2011	0.246	0.814
		2016	0.286	0.000
	Pursat	2011	0.370	0.761
		2017	0.487	0.537
	Ratanakiri	2011	0.442	0.876
		2017	0.526	0.462
		2018	0.562	0.800
	Stung Treng	2018	0.346	0.918
Lao PDR	Attapeu	2011	0.480	0.834
	Champasak	2014	0.000	0.600
		2015	0.504	0.713
	Salavan	2013	0.345	0.822
	Savannakhet	2010	0.500	0.500
		2011	1.000	0.667
Thailand	Ubon Ratchathani	2014	0.366	0.155
		2016	0.495	0.143
		2017	0.533	0.333
		2018	0.000	0.000

**Fig 1:**
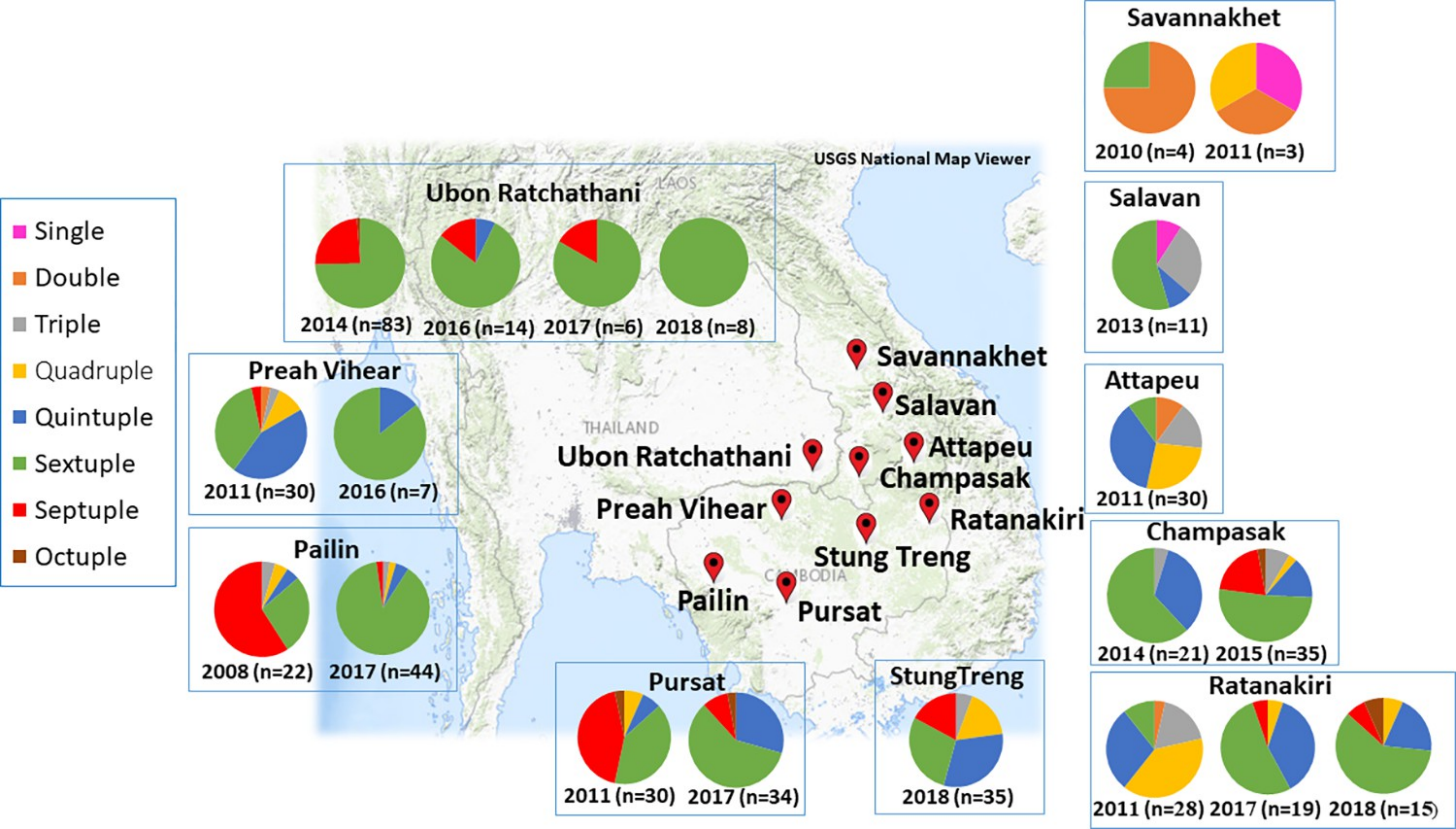
Frequencies of *pfdhfr* and *pfdhps* mutations in Thailand, Cambodia, and Lao PDR between 2008 and 2018.

**Fig 2:**
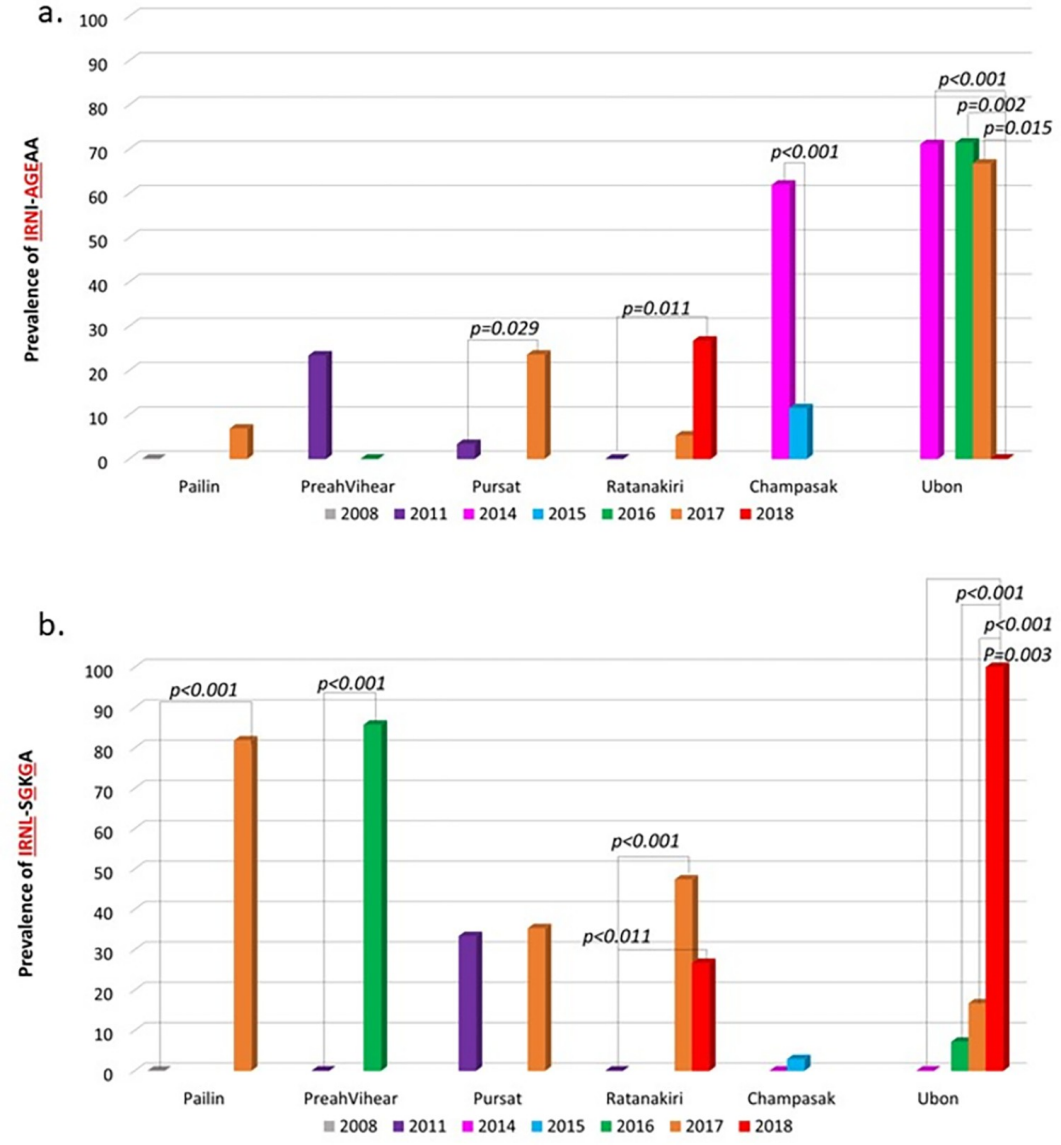
Changes in IRNL-SGKGA and IRNI-AGEAA frequencies of *pfdhfr* and *pfdhps* haplotypes between 2008 and 2018.

**Fig 3:**
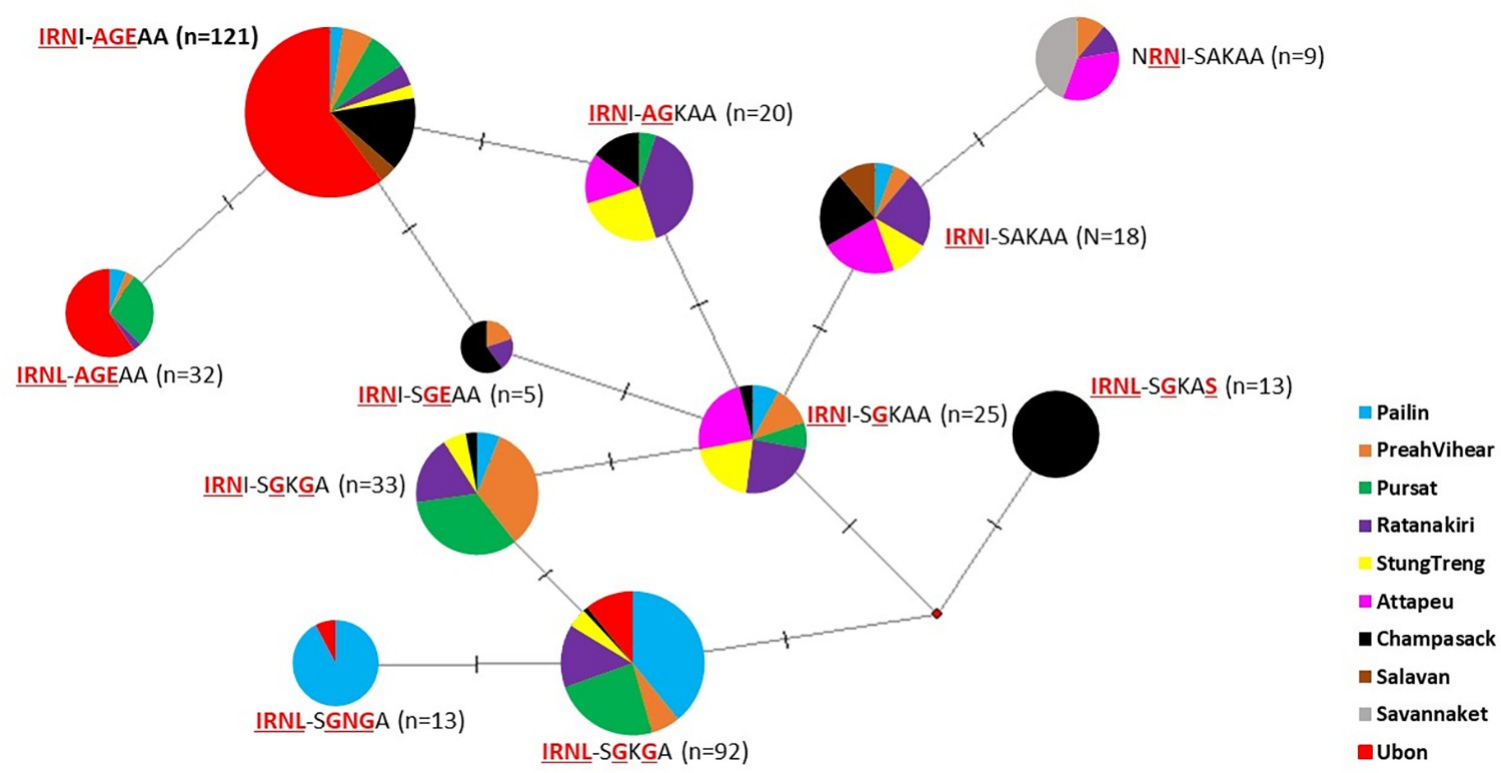
Haplotype network *pfdhfr* and *pfdhps* mutations in Thailand, Cambodia, and Lao PDR for visualizing the relationships among the amino acids within the parasite populations.

Allele combinations with *pfdhfr–pfdhps* alleles were analyzed, including single to octuple mutations (Fig 1). Notably, the current study identified 59 distinct haplotypes (S2 Table). Among them, two major haplotypes of *pfdhfr* and *pfdhps* were observed, namely IRNI-AGEAA (25.31%) and IRNL-SGKGA (19.25%). Across several study sites, including Ubon Ratchathani, Preah Vihear, and Champassak, we observed a reduction in **IRN**I-**AGE**AA but an increase in **IRNL**-S**G**K**G**A (Fig 2). Many study sites have shown an increase in both **IRN**I-**AGE**AA and **IRNL**-S**G**K**G**A, including Pailin, Pursat, and Ratanakiri. The **IRNL**-S**G**K**G**A, which had the highest prevalence observed in the current study, was firstly found in Pursat in 2011. Similarly, octuple mutations were first observed in Pursat in 2011 and then in close study sites, such as Ratanakiri and Champasak by 2018 (S2 Table). Moreover, the dominant haplotypes observed herein were analyzed using the haplotype network for visualizing the relationships among the amino acids within the parasite populations (Fig 3). In the haplotype network (Fig 3), two main haplotypes emerged. The most abundant haplotype, **IRN**I-**AGE**AA, was found in eight different sampling areas. Among several study sites, diversity was largest in Ratanakiri population, as evidenced by both the number of haplotypes found and the distances between them.

The prevalence of *pfdhfr* 51I-59R-108N and *pfdhps* 437G-540E-581G were analyzed and compared with those reported in previous studies available from the online database, WWARN (www.wwarn.org/tracking-resistance/sp-molecular-surveyor). The *pfdhfr* 51I-59R-108N allele was abundantly found in and around Ubon Ratchathani, Pailin, Preah Vihear, Pursat, Ratanakiri, Stung Treng, Attapeu, Champasak, and Salavan (S2 Fig). Conversely, a lower prevalence of the *pfdhfr* 51I-59R-108N allele was observed in Savannakhet. Compared to the results of the *pfdhfr* 51I-59R-108N allele obtained from Chin, Banmauk, and Kayin, Myanmar, those obtained from the current study showed a greater prevalence in the *pfdhfr* 51I-59R-108N allele. In and around Ubon Ratchathani, Pailin, Preah Vihear, Pursat, Ratanakiri, Stung Treng, Attapeu, Champasak, Salavan, and Savannakhet, a low prevalence of *pfdhps* 437G-540E-581G alleles was observed. A high prevalence of the *pfdhps* 437G-540E-581G allele was found in Kayin, Myanmar.

### Prevalence of *pfgch1* gene amplification

In Ubon Ratchathani, Thailand, an increase in *pfgch1* gene amplification was observed from 2016 to 2018 (S1 Fig). *Pfgch1* gene amplification was significantly increased in Ubon Ratchathani, Thailand (p < 0.05) but reduced slightly in Ratanakiri, Cambodia (p = 0.074). In Cambodia, between 2016 and 2018, the *pfgch1* gene were amplified in 14.29%, 63.67%, 47.06%, and 60% of samples from Preah Vihear, Pailin, Pursat, and Stung Treng, respectively. In Champasak, the *pfgch1* gene were amplified in 56.52% of samples collected in 2015. In addition, the association between the *pfgch1* gene amplification and pfdhfr-dhps mutation was analyzed and showed a significant association between the *pfdhps* S436A and K540E and *pfgch1* multiple copies (p<0.05).

## Discussion

Despite having withdrawn SP from Thailand, Cambodia, and Lao PDR for over decade, parasites have still carried the *pfdrfr* and *pfdhps* mutations. Since 2008, evidence from Pailin has shown that parasites carried the *pfdhfr* triple mutations, namely N51I, C59R, and S108N. Moreover, our findings showed an increase in quadruple mutation across Pailin, Preah Vihear, Ubon Ratchathani, Champasak, and Ratanakiri. The increase in quadruple mutations at codons 51, 59, 108, and 164 in *pfdhfr* confers high levels of resistance to PYR [21]. The results obtained herein are consistent with those presented in previous studies, which showed that most *P*. *falciparum* parasites carried triple or quadruple mutations of *pfdhfr* [22, 23]. Mutations in *pfdhfr* attributed to the inhibition constant (Ki) for pyrimethamine had been previously described, suggesting that quadruple mutations, including the I164L position, were critical for the development of pyrimethamine resistance [24–27].

*Pfdhfr* gene mutations observed in the current study revealed a high prevalence of A437G (80%–100%) since 8 to 10 years ago. Notably, S436A and K540E levels were lower in Pailin, Preah Vihear, Pursat, Champasak, Salavan, Savannakhet, and Ubon Ratchathani. When the triple mutation in *pfdhfr* is paired with the double-mutant *pfdhps* (A437G and K540E), the probability of SP treatment failure increases by up to 75% based on previous studies [28, 29]. Moreover, the efficacy can also be strongly affected by host factors.

The current study identified 59 haplotypes using the *pfdhfr* and *pfdhps* genes together. **IRN**I-**AGE**AA and **IRNL**-S**G**K**G**A were the two most common haplotypes found in this investigation. The **IRNL**-S**G**K**G**E haplotype increased over time across several study sites, suggesting a shaping in parasite populations owing to some pressure on parasite populations. There was likely selection of parasites with mutations that can confer resistance to the drugs, leading to drug-selective sweeps. Previously, drug-selective sweeps have been reported to include mutations in *P*. *falciparum* chloroquine resistance transporter (PfCRT) and PfDHFR and PfDHPS, thereby conferring resistance to chloroquine and pyrimethamine/sulfadoxine (PS), respectively [30, 31]. The **IRN**I-**AGE**AA haplotype has three *pfdhfr* point mutations, N51I, C59R, and S108N, as well as a combination of three *pfdhps* point mutations that may result in a high chance of SP resistance or complete resistance [32]. The prevalence of this haplotype was significantly reduced in Champasak and Ubon Ratchathani but increased in Pursat and Ratanakiri. The *Pfdhfr* quadruple mutant **IRNL**-S**G**K**G**A haplotype offers a high level of resistance to PYR [21]. This haplotype was first discovered in Pursat in 2011. Although several study sites have exhibited a rise in this haplotype over time, the current study reported that the two most common haplotypes of *pfdhps* were **AGE**AA and S**G**K**G**A across several study sites. In contrast, a previous study found that the AGEAA triple mutants were predominant in Thailand from 2007 to 2008 albeit being rare S**G**K**G**A mutants [22]. Several factors can explain the persistently high prevalence of antifolate resistance haplotypes observed in the current study, including nature of gene, host/parasite genetic background, drug pressure from non-malarial antifolate drugs, such as trimethoprim and sulfamethoxazole [33] as a part of the standard package of care for people with HIV/AIDS [34, 35]; urinary tract infection [36]; and melioidosis [37]. The combination drug (trimethoprim/sulfamethoxazole) is still wildly and easily available at several local pharmacies throughout the study sites, particularly in Cambodia and Lao PDR. Moreover, there may still be some use of SP outside of the national programs even SP was stopped as national policy in the three countries. In addition, previous reports have shown that human migration contributed to the spread of chloroquine-resistant malaria from southeast Asia to Africa during the 1970s and SP resistance during the 1980s–1990s [38–40]. The high *pfdhfr* and *pfdhps* haplotype diversity in Ratanakiri and Champasak implied a mixture of parasite populations from Lao PDR and Cambodia/Thailand. These results suggest human migration may contribute the SP resistance haplotype from one study site to another. Moreover, octuple mutations were initially discovered in Pursat in 2011 and later in the nearby study locations of Ratanakiri and Champasak in 2015–2018. This octuple mutation combination of quadruple mutations in *pfdhfr* and quadruple mutations in *pfdhps* might confer high levels of resistance to PYR [21, 28, 29]. The *pfgch1* gene amplification in the parasites was investigated and found that 7%-64% of the parasites in Ratanakiri, Preah Vihear, Pailin, Pursat, and Stung Treng had the *pfgch1* gene amplification in 2016–2018. In Ubon Ratchathani, 17%-100% of the parasites had the *pfgch1* gene amplification in 2017–2018 compared with a recent study that found that 8% of the parasites in Ubon Ratchathani had the *pfgch1* gene amplification between 2008 and 2010 [14]. Furthermore, the parasites carry multiple copies of *pfgch1* were also reported in Thai-Myanmar border in 2002–2003 and found that 72% of parasites had the *pfgch1* gene amplification [19]. The precise mechanism of antifolate treatment selected for *pfgch1* gene amplification remains uncertain. There were two possibilities 1) antifolate treatment can directly select for *pfgch1* amplification, and the amplified gch1 parasites may be expected to be more resistant than those with a single copy [19]. Therefore, it may not be expected to see associations with *pfdhfr* or *pfdhps* in the absence of drug selection if this were the case. However, this study and others [19, 41], did observe the association between the *pfdhps* S436A and K540E and *pfgch1* multiple copies, therefore it is less likely. 2) *pfgch1* amplification may compensate for reduced efficacy of *pfdhfr* and/or *pfdhps* enzymes with resistance mutations downstream in the biosynthesis pathway [41]. Although Nair et al. discovered that the associations between *pfdhfr*-164 and *pfgch1* CNP strongly support involvement with *pfdhfr* [19]. Based on our discovery of a significant association between *pfdhps* S436A and K540E and *pfgch1* multiple copies, this supports the idea that *pfgch1* amplification compensates for fitness effects. Even after SP was discontinued, the presence of amplified *pfgch1* was observed exacerbating the resistant problem and may be assisting with maintaining these resistant parasites.

## Conclusion

Despite having withdrawn SP medication from Thailand, Cambodia, and Lao PDR for 10 years, *P*. *falciparum* parasites still contained the *pfdhfr* and *pfdhps* mutations. Among the parasites, several haplotypes were prevalent including **IRN**I-**AGE**AA and **IRNL**-S**G**K**G**A. Moreover, our findings suggested that octuple mutations had recently emerged from Cambodia after determining the prevalence of *pfgch1* gene amplification. Due to the relatively rapid evolution of mutation patterns, these areas require frequent monitoring of *pfdhfr* and *pfdhps*.

## Supporting information

S1 Fig Mapping prevalence of *pfdhfr* (A), *pfdhps* (B) gene, and *pfgch1* (C) gene amplifications.
(PDF)

S2 Fig Prevalence of *pfdhfr* 51I-59R-108N and *pfdhps* 437G-540E-581G. (PDF)

S1 Table Prevalence of *pfdhfr* and *pfdhps* point mutation. (PDF)

S2 Table Haplotypes of *pfdhfr* and *pfdhps* in *Plasmodium falciparum* isolated from Cambodia, Lao PDR, and Thailand. (PDF)

## List of abbreviations

DNA: Deoxyribonucleic acid
PCR: Polymerase Chain Reaction
*P. falciparum*: *Plasmodium falciparum*
*pfdhfr*: *Plasmodium falciparum dihydrofolate reductase*
*pfdhps*: *P. falciparum dihydropteroate synthase*
*pfgch1*: *P. falciparum GTP cyclohydrolase 1*

## References

[1] WHO. The "World malaria report 2019" at a glance. 2019.

[2] Wongsrichanalai C, Sirichaisinthop J, Karwacki JJ, Congpuong K, Miller RS, Pang L, et al. Drug resistant malaria on the Thai-Myanmar and Thai-Cambodian borders. Southeast Asian J Trop Med Public Health. 2001;32(1):41–9. 11485094

[3] Eyles DE, Hoo CC, Warren M, Sandosham AA. Plasmodium Falciparum Resistant to Chloroquine in Cambodia. Am J Trop Med Hyg. 1963;12:840–3. doi: 10.4269/ajtmh.1963.12.840 14072437

[4] Verdrager J. Epidemiology of emergence and spread of drug-resistant falciparum malaria in Southeast Asia. Southeast Asian J Trop Med Public Health. 1986;17(1):111–8. 3526576

[5] WHO. Report on the informal consultation on monitoring resistance to antimalarial drugs in the Mekong Region. World Health Organization, Geneva, Switzerland. 2000.

[6] Wongsrichanalai C, Meshnick SR. Declining artesunate-mefloquine efficacy against falciparum malaria on the Cambodia-Thailand border. Emerg Infect Dis. 2008;14(5):716–9. doi: 10.3201/eid1405.071601 18439351 PMC2600243

[7] Mayxay M, Keomany S, Khanthavong M, Souvannasing P, Stepniewska K, Khomthilath T, et al. A phase III, randomized, non-inferiority trial to assess the efficacy and safety of dihydroartemisinin-piperaquine in comparison with artesunate-mefloquine in patients with uncomplicated Plasmodium falciparum malaria in southern Laos. Am J Trop Med Hyg. 2010;83(6):1221–9. doi: 10.4269/ajtmh.2010.10-0276 21118925 PMC2990035

[8] Triglia T, Wang P, Sims PF, Hyde JE, Cowman AF. Allelic exchange at the endogenous genomic locus in Plasmodium falciparum proves the role of dihydropteroate synthase in sulfadoxine-resistant malaria. EMBO J. 1998;17(14):3807–15. doi: 10.1093/emboj/17.14.3807 9669998 PMC1170716

[9] Koukouikila-Koussounda F, Bakoua D, Fesser A, Nkombo M, Vouvoungui C, Ntoumi F. High prevalence of sulphadoxine-pyrimethamine resistance-associated mutations in Plasmodium falciparum field isolates from pregnant women in Brazzaville, Republic of Congo. Infect Genet Evol. 2015;33:32–6. doi: 10.1016/j.meegid.2015.04.007 25934142

[10] Wang P, Read M, Sims PF, Hyde JE. Sulfadoxine resistance in the human malaria parasite Plasmodium falciparum is determined by mutations in dihydropteroate synthetase and an additional factor associated with folate utilization. Mol Microbiol. 1997;23(5):979–86. doi: 10.1046/j.1365-2958.1997.2821646.x 9076734

[11] Basco LK, Eldin de Pecoulas P, Wilson CM, Le Bras J, Mazabraud A. Point mutations in the dihydrofolate reductase-thymidylate synthase gene and pyrimethamine and cycloguanil resistance in Plasmodium falciparum. Mol Biochem Parasitol. 1995;69(1):135–8. doi: 10.1016/0166-6851(94)00207-4 7723784

[12] Berens N, Schwoebel B, Jordan S, Vanisaveth V, Phetsouvanh R, Christophel EM, et al. Plasmodium falciparum: correlation of in vivo resistance to chloroquine and antifolates with genetic polymorphisms in isolates from the south of Lao PDR. Trop Med Int Health. 2003;8(9):775–82. doi: 10.1046/j.1365-3156.2003.01099.x 12950663

[13] Heinberg A, Siu E, Stern C, Lawrence EA, Ferdig MT, Deitsch KW, et al. Direct evidence for the adaptive role of copy number variation on antifolate susceptibility in Plasmodium falciparum. Mol Microbiol. 2013;88(4):702–12. doi: 10.1111/mmi.12162 23347134 PMC3654098

[14] Sugaram R, Suwannasin K, Kunasol C, Mathema VB, Day NPJ, Sudathip P, et al. Molecular characterization of Plasmodium falciparum antifolate resistance markers in Thailand between 2008 and 2016. Malar J. 2020;19(1):107. doi: 10.1186/s12936-020-03176-x 32127009 PMC7055081

[15] Duraisingh MT, Curtis J, Warhurst DC. Plasmodium falciparum: detection of polymorphisms in the dihydrofolate reductase and dihydropteroate synthetase genes by PCR and restriction digestion. Exp Parasitol. 1998;89(1):1–8. doi: 10.1006/expr.1998.4274 9603482

[16] Parola P, Pradines B, Simon F, Carlotti MP, Minodier P, Ranjeva MP, et al. Antimalarial drug susceptibility and point mutations associated with drug resistance in 248 Plasmodium falciparum isolates imported from Comoros to Marseille, France in 2004 2006. Am J Trop Med Hyg. 2007;77(3):431–7. 17827355

[17] Tanomsing N, Imwong M, Pukrittayakamee S, Chotivanich K, Looareesuwan S, Mayxay M, et al. Genetic analysis of the dihydrofolate reductase-thymidylate synthase gene from geographically diverse isolates of Plasmodium malariae. Antimicrob Agents Chemother. 2007;51(10):3523–30. doi: 10.1128/AAC.00234-07 17682097 PMC2043249

[18] Vinayak S, Alam MT, Mixson-Hayden T, McCollum AM, Sem R, Shah NK, et al. Origin and evolution of sulfadoxine resistant Plasmodium falciparum. PLoS Pathog. 2010;6(3):e1000830. doi: 10.1371/journal.ppat.1000830 20360965 PMC2847944

[19] Nair S, Miller B, Barends M, Jaidee A, Patel J, Mayxay M, et al. Adaptive copy number evolution in malaria parasites. PLoS Genet. 2008;4(10):e1000243. doi: 10.1371/journal.pgen.1000243 18974876 PMC2570623

[20] M N. Molecular evolutionary genetics. New York: Colombia University Press. 512 p. 1987.

[21] Kiara SM, Okombo J, Masseno V, Mwai L, Ochola I, Borrmann S, et al. In vitro activity of antifolate and polymorphism in dihydrofolate reductase of Plasmodium falciparum isolates from the Kenyan coast: emergence of parasites with Ile-164-Leu mutation. Antimicrob Agents Chemother. 2009;53(9):3793–8. doi: 10.1128/AAC.00308-09 19528269 PMC2737895

[22] Alam MT, Vinayak S, Congpuong K, Wongsrichanalai C, Satimai W, Slutsker L, et al. Tracking origins and spread of sulfadoxine-resistant Plasmodium falciparum dhps alleles in Thailand. Antimicrob Agents Chemother. 2011;55(1):155–64. doi: 10.1128/AAC.00691-10 20956597 PMC3019650

[23] Imwong M, Jindakhad T, Kunasol C, Sutawong K, Vejakama P, Dondorp AM. An outbreak of artemisinin resistant falciparum malaria in Eastern Thailand. Sci Rep. 2015;5:17412. doi: 10.1038/srep17412 26616851 PMC4663761

[24] Sirawaraporn W, Sathitkul T, Sirawaraporn R, Yuthavong Y, Santi DV. Antifolate-resistant mutants of Plasmodium falciparum dihydrofolate reductase. Proc Natl Acad Sci U S A. 1997;94(4):1124–9. doi: 10.1073/pnas.94.4.1124 9037017 PMC19755

[25] Cowman AF, Morry MJ, Biggs BA, Cross GA, Foote SJ. Amino acid changes linked to pyrimethamine resistance in the dihydrofolate reductase-thymidylate synthase gene of Plasmodium falciparum. Proc Natl Acad Sci U S A. 1988;85(23):9109–13. doi: 10.1073/pnas.85.23.9109 3057499 PMC282673

[26] Peterson DS, Walliker D, Wellems TE. Evidence that a point mutation in dihydrofolate reductase-thymidylate synthase confers resistance to pyrimethamine in falciparum malaria. Proc Natl Acad Sci U S A. 1988;85(23):9114–8. doi: 10.1073/pnas.85.23.9114 2904149 PMC282674

[27] Thaithong S, Chan SW, Songsomboon S, Wilairat P, Seesod N, Sueblinwong T, et al. Pyrimethamine resistant mutations in Plasmodium falciparum. Mol Biochem Parasitol. 1992;52(2):149–57. doi: 10.1016/0166-6851(92)90047-n 1620155

[28] Staedke SG, Sendagire H, Lamola S, Kamya MR, Dorsey G, Rosenthal PJ. Relationship between age, molecular markers, and response to sulphadoxine-pyrimethamine treatment in Kampala, Uganda. Trop Med Int Health. 2004;9(5):624–9. doi: 10.1111/j.1365-3156.2004.01239.x 15117308

[29] Kublin JG, Dzinjalamala FK, Kamwendo DD, Malkin EM, Cortese JF, Martino LM, et al. Molecular markers for failure of sulfadoxine-pyrimethamine and chlorproguanil-dapsone treatment of Plasmodium falciparum malaria. J Infect Dis. 2002;185(3):380–8. doi: 10.1086/338566 11807721

[30] Peterson DS, Milhous WK, Wellems TE. Molecular basis of differential resistance to cycloguanil and pyrimethamine in Plasmodium falciparum malaria. Proc Natl Acad Sci U S A. 1990;87(8):3018–22. doi: 10.1073/pnas.87.8.3018 2183222 PMC53825

[31] Fidock DA, Nomura T, Talley AK, Cooper RA, Dzekunov SM, Ferdig MT, et al. Mutations in the P. falciparum digestive vacuole transmembrane protein PfCRT and evidence for their role in chloroquine resistance. Mol Cell. 2000;6(4):861–71. doi: 10.1016/s1097-2765(05)00077-8 11090624 PMC2944663

[32] Chaturvedi R, Chhibber-Goel J, Verma I, Gopinathan S, Parvez S, Sharma A. Geographical spread and structural basis of sulfadoxine-pyrimethamine drug-resistant malaria parasites. Int J Parasitol. 2021;51(7):505–25. doi: 10.1016/j.ijpara.2020.12.011 33775670

[33] Iyer JK, Milhous WK, Cortese JF, Kublin JG, Plowe CV. Plasmodium falciparum cross-resistance between trimethoprim and pyrimethamine. Lancet. 2001;358(9287):1066–7. doi: 10.1016/S0140-6736(01)06201-8 11589941

[34] Chintu C, Bhat GJ, Walker AS, Mulenga V, Sinyinza F, Lishimpi K, et al. Co-trimoxazole as prophylaxis against opportunistic infections in HIV-infected Zambian children (CHAP): a double-blind randomised placebo-controlled trial. Lancet. 2004;364(9448):1865–71. doi: 10.1016/S0140-6736(04)17442-4 15555666

[35] Walker AS, Ford D, Gilks CF, Munderi P, Ssali F, Reid A, et al. Daily co-trimoxazole prophylaxis in severely immunosuppressed HIV-infected adults in Africa started on combination antiretroviral therapy: an observational analysis of the DART cohort. Lancet. 2010;375(9722):1278–86. doi: 10.1016/S0140-6736(10)60057-8 20347483 PMC2858802

[36] Guneysel O, Onur O, Erdede M, Denizbasi A. Trimethoprim/sulfamethoxazole resistance in urinary tract infections. J Emerg Med. 2009;36(4):338–41. doi: 10.1016/j.jemermed.2007.08.068 18325714

[37] Cheng AC, McBryde ES, Wuthiekanun V, Chierakul W, Amornchai P, Day NP, et al. Dosing regimens of cotrimoxazole (trimethoprim-sulfamethoxazole) for melioidosis. Antimicrob Agents Chemother. 2009;53(10):4193–9. doi: 10.1128/AAC.01301-08 19620336 PMC2764189

[38] Anderson TJ, Roper C. The origins and spread of antimalarial drug resistance: lessons for policy makers. Acta Trop. 2005;94(3):269–80. doi: 10.1016/j.actatropica.2005.04.010 15878153

[39] Naidoo I, Roper C. Following the path of most resistance: dhps K540E dispersal in African Plasmodium falciparum. Trends Parasitol. 2010;26(9):447–56. doi: 10.1016/j.pt.2010.05.001 20728060

[40] Martens P, Hall L. Malaria on the move: human population movement and malaria transmission. Emerg Infect Dis. 2000;6(2):103–9. doi: 10.3201/eid0602.000202 10756143 PMC2640853

[41] Kidgell C, Volkman SK, Daily J, Borevitz JO, Plouffe D, Zhou Y, et al. A systematic map of genetic variation in Plasmodium falciparum. PLoS Pathog. 2006;2(6):e57. doi: 10.1371/journal.ppat.0020057 16789840 PMC1480597

