# Supplementary material for: Ten-year persistence and evolution of Plasmodium falciparum antifolate and anti-sulfonamide resistance markers pfdhfr and pfdhps in three Asian countries
Source: PLoS One. 2022 Dec 16;17(12):e0278928. doi: 10.1371/journal.pone.0278928 (PMC9757559; doi:10.1371/journal.pone.0278928)
Supplement: S1 Fig — Mapping prevalence of pfdhfr (A), pfdhps (B) gene, and pfgch1 (C) gene amplifications. (PDF) [file pone.0278928.s001.pdf]

Mapping prevalence of *pfdhfr*(A), *pfdhps*(B) gene, and *pfgch1*(C) gene amplifications.

A

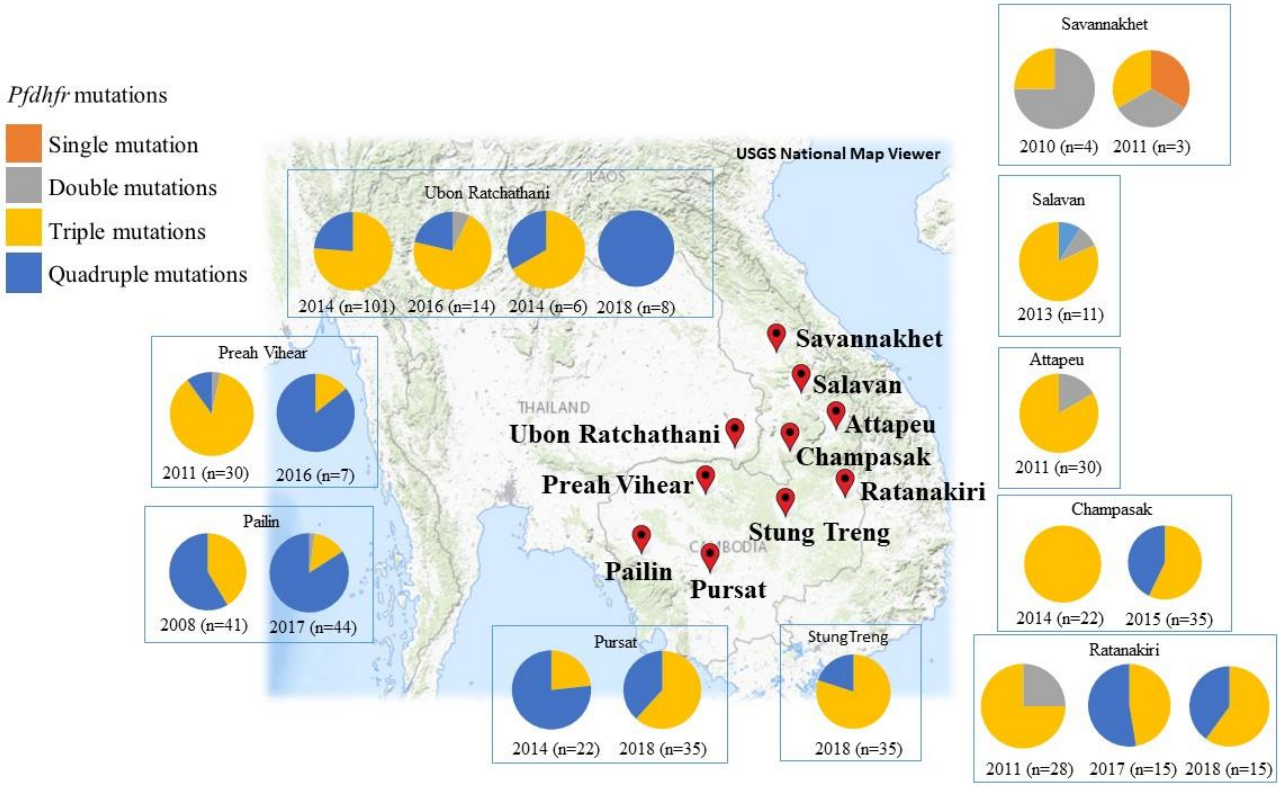

B

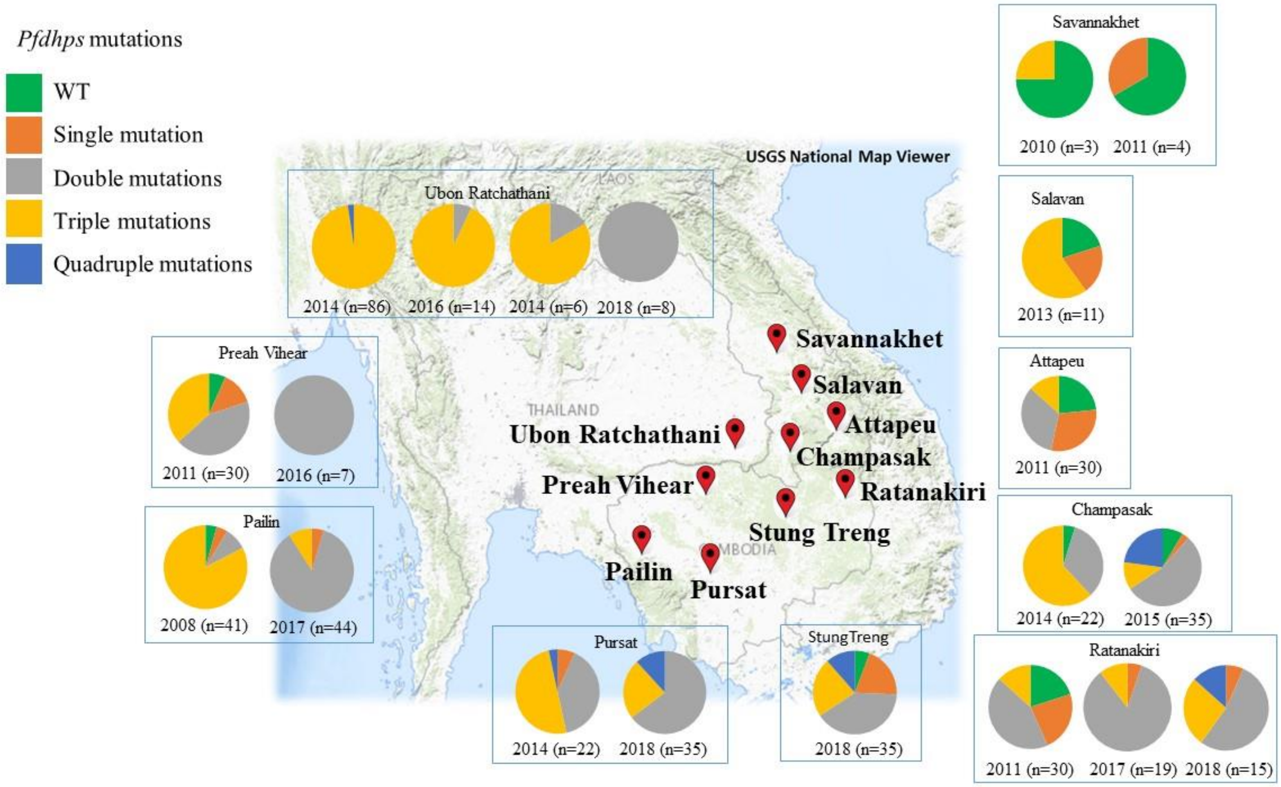

C

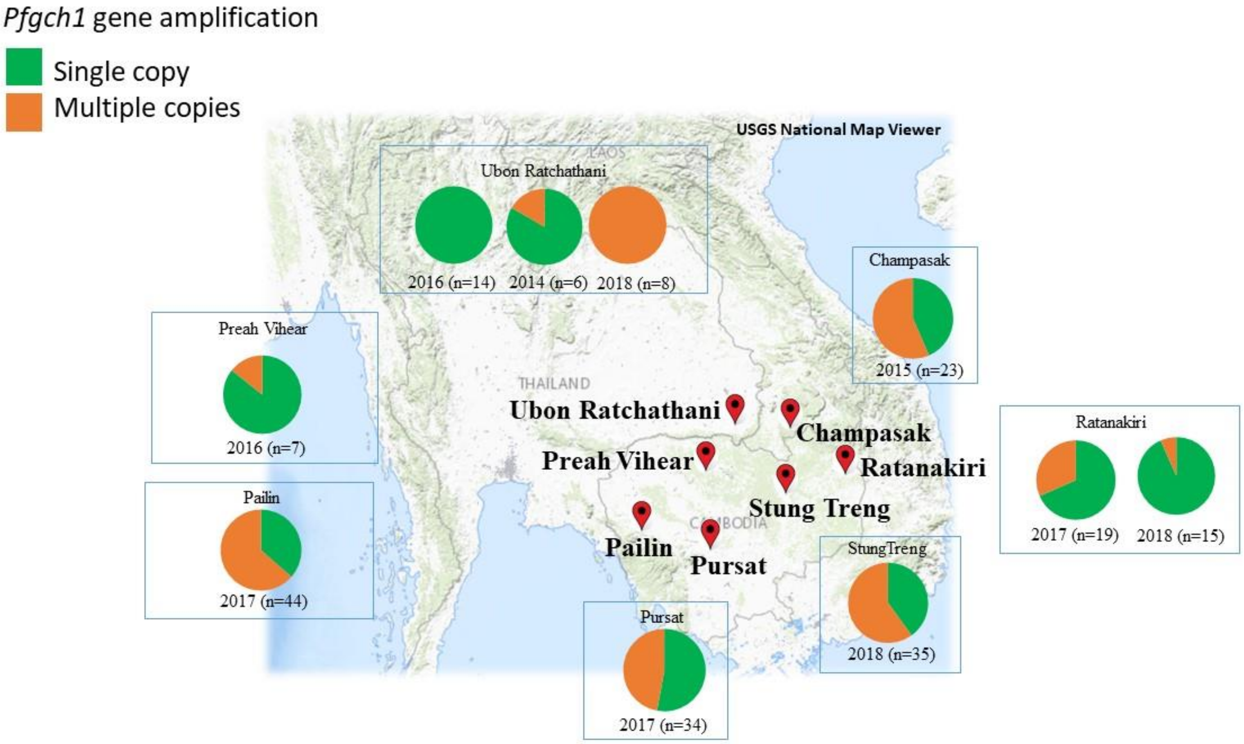
Pfgch1 gene amplification
Single copy
Multiple copies
USGS National Map Viewer
Ubon Ratchathani
2016 (n=14)
2014 (n=6)
2018 (n=8)
Champasak
2015 (n=23)
Preah Vihear
2016 (n=7)
Pailin
2017 (n=44)
Ratanakiri
2017 (n=19)
2018 (n=15)
StungTreng
2018 (n=35)
Pursat
2017 (n=34)
THAILAND
CAMBODIA
LAOS
Ubon Ratchathani
Champasak
Preah Vihear
Ratanakiri
Stung Treng
Pailin
Pursat
