# Supplementary figures and images for: Ten-year persistence and evolution of Plasmodium falciparum antifolate and anti-sulfonamide resistance markers pfdhfr and pfdhps in three Asian countries
Source: PLoS One. 2022 Dec 16;17(12):e0278928. doi: 10.1371/journal.pone.0278928 (PMC9757559; doi:10.1371/journal.pone.0278928)

Prevalence of *pfdhfr* 51I-59R-108N (a.) and *pfdhps* 437G-540E-581G (b.)

a.

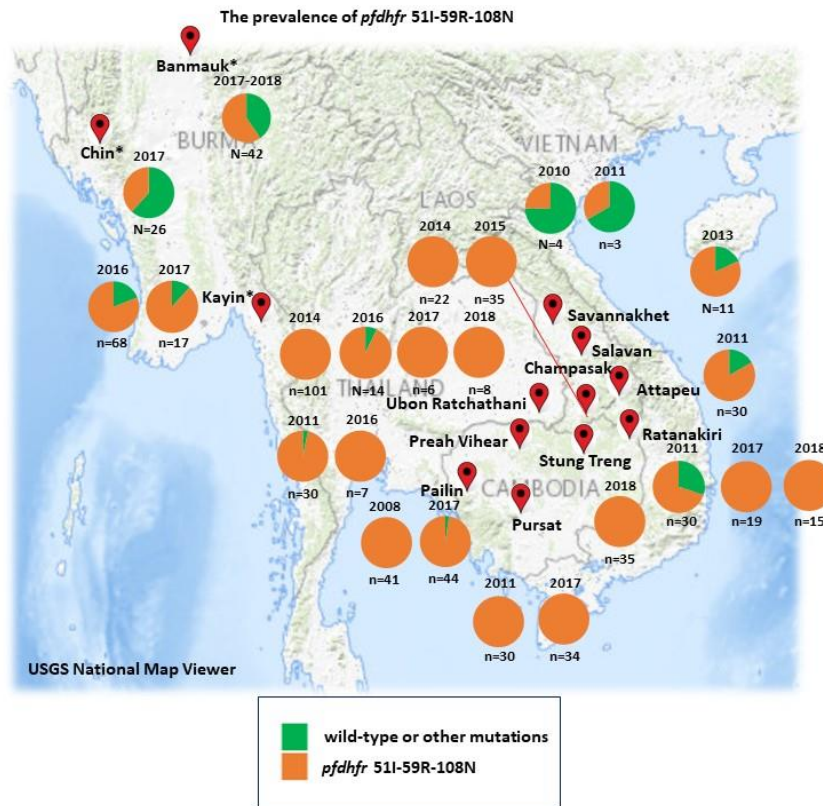

b.

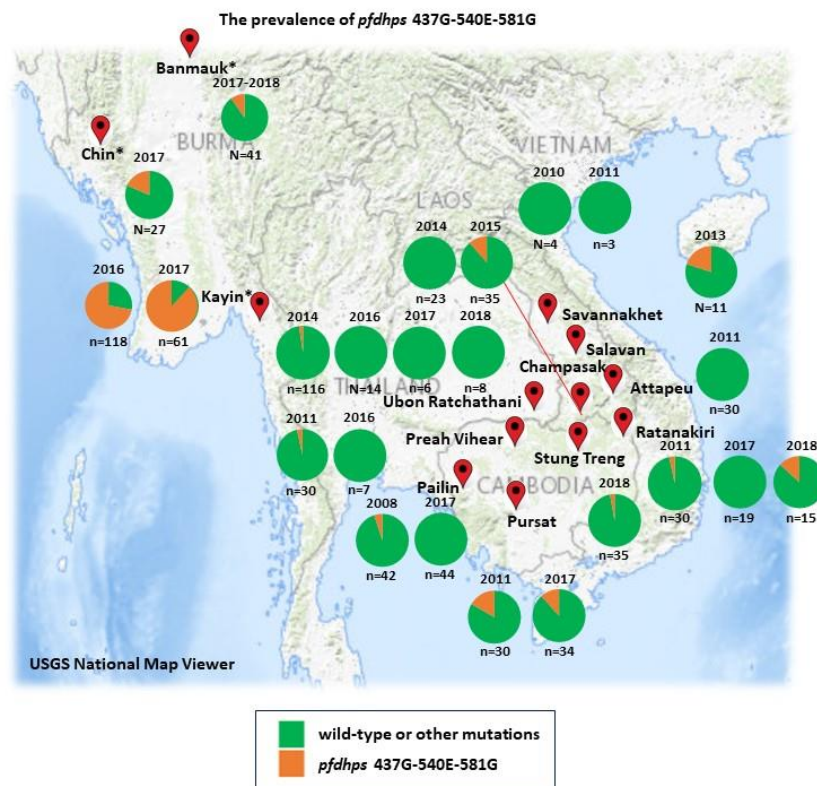

Supplement: S2 Fig — (PDF) [file pone.0278928.s002.pdf]
