# Supplementary material for: Ten-year persistence and evolution of Plasmodium falciparum antifolate and anti-sulfonamide resistance markers pfdhfr and pfdhps in three Asian countries
Source: PLoS One. 2022 Dec 16;17(12):e0278928. doi: 10.1371/journal.pone.0278928 (PMC9757559; doi:10.1371/journal.pone.0278928)
Supplement: S1 Table — (PDF) [file pone.0278928.s003.pdf]

Table: Prevalence of *pfdhfr* point mutations

Countries	Study sites	Study Years	Number of Samples	*pfdhfr* N51I (%)		C59R(%)		S108N(%)		I164L(%)	
				Mutant	Mixed	Mutant	Mixed	Mutant	Mixed	Mutant	Mixed
Cambodia	Pailin	2008	41	100	0	100	0	100	0	59	0
	Pailin	2017	44	100	0	98	0	100	0	84	0
	PreahVihear	2011	30	97	0	100	0	100	0	10	0
	PreahVihear	2016	7	100	0	100	0	100	0	86	0
	Pursat	2011	30	100	0	100	0	100	0	77	0
	Pursat	2017	34	100	0	100	0	100	0	38	0
	Ratanakiri	2011	28	71	4	100	0	100	0	0	0
	Ratanakiri	2017	19	100	0	100	0	100	0	53	0
	Ratanakiri	2018	15	100	0	100	0	100	0	33	7
	StungTreng	2018	35	100	0	100	0	100	0	14	6
Lao PDR	Attapeu	2011	30	70	13	100	0	100	0	0	0
	Champasak	2014	22	100	0	100	0	100	0	0	0
	Champasak	2015	35	100	0	100	0	100	0	43	0
	Salavan	2013	11	82	0	91	0	91	0	0	0
	Savannaket	2010	4	0	25	100	0	100	0	0	0
	Savannaket	2011	3	0	33	67	0	67	2	0	0
Thailand	Ubon	2014	101	100	0	100	0	100	0	24	0
	Ubon	2016	14	93	0	100	0	100	0	14	7
	Ubon	2017	6	100	0	100	0	100	0	33	0
	Ubon	2018	8	100	0	100	0	100	0	100	0

The colors were highlighted based on the prevalence

Color	Prevalence
Green	0%
Yellow	1-25%
Light orange	26-50%
Orange	51-75%
Red	76-100%

Table: Prevalence of *pfdhps* point mutations

Countries	Study sites	Study Years	*pfdhps*										
			Number of Samples	S436A(%)		A437G(%)		K540E(%)		A581G(%)		A613S(%)	
				Mutant	Mixed	Mutant	Mixed	Mutant	Mixed	Mutant	Mixed	Mutant	Mixed
Cambodia	Pailin	2008	23	4	0	91	4	87	4	78	0	0	0
	Pailin	2017	44	9	0	100	0	9	0	86	0	0	0
	PreahVihear	2011	30	30	3	93	0	33	10	33	7	0	0
	PreahVihear	2016	7	0	0	100	0	0	0	100	0	0	0
	Pursat	2011	30	40	7	100	0	43	7	53	3	0	0
	Pursat	2017	34	24	12	100	0	26	9	71	6	0	0
	Ratanakiri	2011	30	37	0	70	3	0	23	3	13	0	0
	Ratanakiri	2017	19	16	0	100	0	16	0	74	0	0	0
	Ratanakiri	2018	15	40	7	100	0	33	7	40	20	0	0
	StungTreng	2018	35	34	17	80	11	23	9	20	11	9	0
Lao PDR	Attapeu	2011	30	23	13	73	3	0	23	0	0	0	0
	Champasak	2014	21	76	0	95	0	76	0	5	0	0	0
	Champasak	2015	35	31	3	91	0	31	3	14	0	63	0
	Salavan	2013	10	40	0	80	0	50	10	20	0	0	0
	Savannaket	2010	4	25	0	25	0	25	0	0	0	0	0
	Savannaket	2011	3	0	0	33	0	0	0	0	0	0	0
Thailand	Ubon	2014	86	94	0	100	0	100	0	8	0	0	0
	Ubon	2016	14	93	0	100	0	93	0	7	0	0	0
	Ubon	2017	6	83	0	100	0	83	0	17	0	0	0
	Ubon	2018	8	0	0	100	0	0	0	100	0	0	0

The colors were highlighted based on the prevalence

Color	Prevalence
Green	0%
Yellow	1-25%
Light orange	26-50%
Orange	51-75%
Red	76-100%
