# Supplementary material for: Ten-year persistence and evolution of Plasmodium falciparum antifolate and anti-sulfonamide resistance markers pfdhfr and pfdhps in three Asian countries
Source: PLoS One. 2022 Dec 16;17(12):e0278928. doi: 10.1371/journal.pone.0278928 (PMC9757559; doi:10.1371/journal.pone.0278928)
Supplement: S2 Table — (PDF) [file pone.0278928.s004.pdf]

Haplotypes of *pfdhfr-pfdhps* of *Plasmodium falciparum* isolated from Cambodia, Laos, and Thailand.

		Prevalence of Haplotypes (%)																			
		Cambodia										Lao PDR						Thailand			
Mutations	Haplotypes	Pailin		PreahVihear		Pursat		Ratanakiri			Stung Treng	Attapeu	Champasak		Salavan	Savannaket		Ubon			
		2008 (%)	2017(%)	2011(%)	2016(%)	2011 (%)	2017(%)	2011(%)	2017(%)	2018(%)	2018(%)	2011(%)	2014(%)	2015(%)	2013(%)	2010(%)	2011(%)	2014(%)	2016(%)	2017(%)	2018(%)
		n=22	n=44	n=30	n=7	n=30	n=34	n=28	n=19	n=15	n=35	n=30	n=21	n=35	n=10	n=4	n=3	n=83	n=14	n=6	n=8
Single	NCS/NI-SAKAA	0.00	0.00	0.00	0.00	0.00	0.00	0.00	0.00	0.00	0.00	0.00	0.00	0.00	0.00	0.00	33.33	0.00	0.00	0.00	0.00
	NCSI-SGKAA	0.00	0.00	0.00	0.00	0.00	0.00	0.00	0.00	0.00	0.00	0.00	0.00	0.00	10.00	0.00	0.00	0.00	0.00	0.00	0.00
Double	NRNI-SAKAA	0.00	0.00	3.33	0.00	0.00	0.00	3.57	0.00	0.00	0.00	10.00	0.00	0.00	0.00	75.00	33.33	0.00	0.00	0.00	0.00
Triple	ICNI-SGKAA	0.00	2.27	0.00	0.00	0.00	0.00	0.00	0.00	0.00	0.00	0.00	0.00	0.00	0.00	0.00	0.00	0.00	0.00	0.00	0.00
	IRNI-SAKAA	4.55	0.00	3.33	0.00	0.00	0.00	14.29	0.00	0.00	5.71	13.33	4.76	8.57	20.00	0.00	0.00	0.00	0.00	0.00	0.00
	NRNI-SGKAA	0.00	0.00	0.00	0.00	0.00	0.00	3.57	0.00	0.00	0.00	3.33	0.00	0.00	10.00	0.00	0.00	0.00	0.00	0.00	0.00
Quadruple	IRNI-AAKAA	0.00	0.00	0.00	0.00	0.00	0.00	3.57	0.00	0.00	0.00	0.00	0.00	0.00	0.00	0.00	0.00	0.00	0.00	0.00	0.00
	IRNI-S/AAKAA	0.00	0.00	0.00	0.00	0.00	0.00	0.00	0.00	0.00	2.86	0.00	0.00	0.00	0.00	0.00	0.00	0.00	0.00	0.00	0.00
	IRNI-SAK/EAA	0.00	0.00	0.00	0.00	0.00	0.00	3.57	0.00	0.00	0.00	0.00	0.00	0.00	0.00	0.00	0.00	0.00	0.00	0.00	0.00
	IRNI-SGKAA	4.55	2.27	10.00	0.00	6.67	0.00	14.29	5.26	6.67	14.29	20.00	0.00	2.86	0.00	0.00	0.00	0.00	0.00	0.00	0.00
	N/IRNI-SGKAA	0.00	0.00	0.00	0.00	0.00	0.00	0.00	0.00	0.00	0.00	6.67	0.00	0.00	0.00	0.00	33.33	0.00	0.00	0.00	0.00
	NRNI-AGKAA	0.00	0.00	0.00	0.00	0.00	0.00	3.57	0.00	0.00	0.00	0.00	0.00	0.00	0.00	0.00	0.00	0.00	0.00	0.00	0.00
	NRNI-SGKA/GA	0.00	0.00	0.00	0.00	0.00	0.00	14.29	0.00	0.00	0.00	0.00	0.00	0.00	0.00	0.00	0.00	0.00	0.00	0.00	0.00
Quintuple	IRNI-AGKAA	0.00	0.00	0.00	0.00	3.33	0.00	21.43	5.26	6.67	14.29	10.00	14.29	0.00	0.00	0.00	0.00	0.00	0.00	0.00	0.00
	IRNI-S/AGKAA	0.00	0.00	0.00	0.00	0.00	0.00	0.00	0.00	0.00	0.00	6.67	0.00	0.00	0.00	0.00	0.00	0.00	0.00	0.00	0.00
	IRNI-SA/GK/EAA	4.55	0.00	0.00	0.00	0.00	0.00	0.00	0.00	0.00	0.00	0.00	0.00	0.00	0.00	0.00	0.00	0.00	0.00	0.00	0.00
	IRNI-SA/GKA/GA	0.00	0.00	0.00	0.00	0.00	0.00	0.00	0.00	0.00	2.86	0.00	0.00	0.00	0.00	0.00	0.00	0.00	0.00	0.00	0.00
	IRNI-SGEAA	0.00	0.00	3.33	0.00	0.00	0.00	0.00	5.26	0.00	0.00	0.00	14.29	0.00	0.00	0.00	0.00	0.00	0.00	0.00	0.00
	IRNI-SGK/EAA	0.00	0.00	0.00	0.00	0.00	0.00	3.57	0.00	0.00	0.00	10.00	0.00	0.00	0.00	0.00	0.00	0.00	0.00	0.00	0.00
	IRNI-SGKA/GA	0.00	0.00	3.33	0.00	0.00	0.00	0.00	0.00	6.67	5.71	0.00	0.00	0.00	0.00	0.00	0.00	0.00	0.00	0.00	0.00
	IRNI-SGKAS	0.00	0.00	0.00	0.00	0.00	0.00	0.00	0.00	0.00	0.00	0.00	0.00	14.29	0.00	0.00	0.00	0.00	0.00	0.00	0.00
	IRNI-SGKGA	0.00	4.55	33.33	14.29	3.33	29.41	0.00	26.32	6.67	5.71	0.00	4.76	0.00	0.00	0.00	0.00	0.00	0.00	0.00	0.00
	IRNL-SGKAA	0.00	0.00	3.33	0.00	0.00	0.00	0.00	0.00	0.00	2.86	0.00	0.00	0.00	0.00	0.00	0.00	0.00	0.00	0.00	0.00
	N/IRNI-S/AA/GKAA	0.00	0.00	0.00	0.00	0.00	0.00	0.00	0.00	0.00	0.00	3.33	0.00	0.00	0.00	0.00	0.00	0.00	0.00	0.00	0.00
	N/IRNI-S/AGKAA	0.00	0.00	0.00	0.00	0.00	0.00	0.00	0.00	0.00	0.00	3.33	0.00	0.00	0.00	0.00	0.00	0.00	0.00	0.00	0.00
	N/IRNI-SGK/EAA	0.00	0.00	0.00	0.00	0.00	0.00	3.57	0.00	0.00	0.00	0.00	0.00	0.00	0.00	0.00	0.00	0.00	0.00	0.00	0.00
	NRNI-AGEAA	0.00	0.00	0.00	0.00	0.00	0.00	0.00	0.00	0.00	0.00	0.00	0.00	0.00	0.00	0.00	0.00	0.00	7.14	0.00	0.00
	NRNI-AGK/EAA	0.00	0.00	0.00	0.00	0.00	0.00	0.00	0.00	0.00	0.00	3.33	0.00	0.00	0.00	0.00	0.00	0.00	0.00	0.00	0.00

The colors were highlighted based on the prevalence

Colour	Prevalence
Green	0%
Yellow	1-25%
Light orange	26-50%
Orange	51-75%
Red	76-100%

Haplotypes of *pfdhfr-pfdhps* of *Plasmodium falciparum* isolated from Cambodia, Laos, and Thailand.

		Prevalence of Haplotypes (%)																			
		Cambodia										Lao PDR						Thailand			
Mutations	Haplotypes	Pailin		PreahVihear		Pursat		Ratanakiri			Stung Treng	Attapeu	Champasak		Salavan	Savannaket		Ubon			
		2008 (%)	2017(%)	2011(%)	2016(%)	2011 (%)	2017(%)	2011(%)	2017(%)	2018(%)	2018(%)	2011(%)	2014(%)	2015(%)	2013(%)	2010(%)	2011(%)	2014(%)	2016(%)	2017(%)	2018(%)
		n=22	n=44	n=30	n=7	n=30	n=34	n=28	n=19	n=15	n=35	n=30	n=21	n=35	n=10	n=4	n=3	n=83	n=14	n=6	n=8
Sextuple	IRNI-AGEAA	0.00	6.82	23.33	0.00	3.33	23.53	0.00	5.26	26.67	8.57	0.00	61.90	11.43	40.00	0.00	0.00	71.08	71.43	66.67	0.00
	IRNI-AGK/EAA	0.00	0.00	3.33	0.00	0.00	0.00	3.57	0.00	0.00	0.00	10.00	0.00	0.00	0.00	0.00	0.00	0.00	0.00	0.00	0.00
	IRNI-S/AA/GK/EAA	0.00	0.00	0.00	0.00	0.00	0.00	0.00	0.00	0.00	2.86	0.00	0.00	0.00	0.00	0.00	0.00	0.00	0.00	0.00	0.00
	IRNI-S/AGK/EAA	0.00	0.00	3.33	0.00	0.00	0.00	0.00	0.00	0.00	2.86	0.00	0.00	0.00	0.00	0.00	0.00	0.00	0.00	0.00	0.00
	IRNI-S/AGKGA	0.00	0.00	0.00	0.00	3.33	0.00	0.00	0.00	0.00	2.86	0.00	0.00	0.00	0.00	0.00	0.00	0.00	0.00	0.00	0.00
	IRNI-SGEGA	0.00	0.00	0.00	0.00	0.00	0.00	0.00	0.00	0.00	0.00	0.00	0.00	0.00	10.00	0.00	0.00	0.00	0.00	0.00	0.00
	IRNI-SGK/EA/GA	0.00	0.00	3.33	0.00	0.00	0.00	0.00	0.00	0.00	0.00	0.00	0.00	0.00	0.00	0.00	0.00	0.00	0.00	0.00	0.00
	IRNI-SGK/EGA	0.00	0.00	0.00	0.00	0.00	0.00	3.57	0.00	0.00	0.00	0.00	0.00	0.00	10.00	0.00	0.00	0.00	0.00	0.00	0.00
	IRNI-SGNGA	22.73	0.00	0.00	0.00	0.00	0.00	0.00	0.00	0.00	0.00	0.00	0.00	0.00	0.00	0.00	0.00	3.61	0.00	0.00	0.00
	IRNI/L-SGKGA	0.00	0.00	0.00	0.00	0.00	0.00	0.00	0.00	6.67	0.00	0.00	0.00	0.00	0.00	0.00	0.00	0.00	0.00	0.00	0.00
	IRNL-SGEAA	4.55	0.00	3.33	0.00	0.00	0.00	0.00	0.00	0.00	0.00	0.00	0.00	0.00	0.00	0.00	0.00	0.00	0.00	0.00	0.00
	IRNL-SGKAS	0.00	0.00	0.00	0.00	0.00	0.00	0.00	0.00	0.00	0.00	0.00	0.00	37.14	0.00	0.00	0.00	0.00	0.00	0.00	0.00
	IRNL-SGKGA	0.00	81.82	0.00	85.71	33.33	35.29	0.00	47.37	26.67	11.43	0.00	0.00	2.86	0.00	0.00	0.00	0.00	7.14	16.67	100.00
	N/IRNI-AGEAA	0.00	0.00	0.00	0.00	0.00	0.00	0.00	0.00	0.00	0.00	0.00	0.00	0.00	0.00	25.00	0.00	0.00	0.00	0.00	0.00
Septuple	IRNI-AGEA/GA	0.00	0.00	0.00	0.00	0.00	0.00	0.00	0.00	6.67	0.00	0.00	0.00	0.00	0.00	0.00	0.00	0.00	0.00	0.00	0.00
	IRNI-AGEAS	0.00	0.00	0.00	0.00	0.00	0.00	0.00	0.00	0.00	8.57	0.00	0.00	8.57	0.00	0.00	0.00	0.00	0.00	0.00	0.00
	IRNI-AGEGA	0.00	0.00	0.00	0.00	0.00	0.00	0.00	0.00	0.00	0.00	0.00	0.00	11.43	0.00	0.00	0.00	1.20	0.00	0.00	0.00
	IRNI-AGK/EA/GA	0.00	0.00	0.00	0.00	3.33	0.00	0.00	0.00	0.00	2.86	0.00	0.00	0.00	0.00	0.00	0.00	0.00	0.00	0.00	0.00
	IRNI-S/AGK/EA/GA	0.00	0.00	0.00	0.00	0.00	2.94	0.00	0.00	0.00	0.00	0.00	0.00	0.00	0.00	0.00	0.00	0.00	0.00	0.00	0.00
	IRNI-S/AGK/EGA	0.00	0.00	0.00	0.00	0.00	5.88	0.00	0.00	0.00	0.00	0.00	0.00	0.00	0.00	0.00	0.00	0.00	0.00	0.00	0.00
	IRNI/L-AGEAA	0.00	0.00	0.00	0.00	0.00	0.00	0.00	0.00	0.00	0.00	0.00	0.00	0.00	0.00	0.00	0.00	0.00	7.14	0.00	0.00
	IRNI/L-S/AA/GEAA	0.00	0.00	0.00	0.00	0.00	0.00	0.00	0.00	0.00	5.71	0.00	0.00	0.00	0.00	0.00	0.00	0.00	0.00	0.00	0.00
	IRNL-AGEAA	4.55	2.27	3.33	0.00	30.00	0.00	0.00	5.26	0.00	0.00	0.00	0.00	0.00	0.00	0.00	0.00	20.48	7.14	16.67	0.00
	IRNL-SGEGA	0.00	0.00	0.00	0.00	10.00	0.00	0.00	0.00	0.00	0.00	0.00	0.00	0.00	0.00	0.00	0.00	1.20	0.00	0.00	0.00
	IRNL-SGNGA	54.55	0.00	0.00	0.00	0.00	0.00	0.00	0.00	0.00	0.00	0.00	0.00	0.00	0.00	0.00	0.00	1.20	0.00	0.00	0.00
Octuple	IRNL-AGNGA	0.00	0.00	0.00	0.00	0.00	0.00	0.00	0.00	0.00	0.00	0.00	0.00	0.00	0.00	0.00	0.00	1.20	0.00	0.00	0.00
	IRNL-S/AGEA/GA	0.00	0.00	0.00	0.00	0.00	2.94	0.00	0.00	0.00	0.00	0.00	0.00	0.00	0.00	0.00	0.00	0.00	0.00	0.00	0.00
	IRNL-S/AGK/EA/GA	0.00	0.00	0.00	0.00	0.00	0.00	0.00	0.00	6.67	0.00	0.00	0.00	0.00	0.00	0.00	0.00	0.00	0.00	0.00	0.00
	IRNL-S/AGK/EAS	0.00	0.00	0.00	0.00	0.00	0.00	0.00	0.00	0.00	0.00	0.00	0.00	2.86	0.00	0.00	0.00	0.00	0.00	0.00	0.00
	IRNL-S/AGK/EGA	0.00	0.00	0.00	0.00	3.33	0.00	0.00	0.00	0.00	0.00	0.00	0.00	0.00	0.00	0.00	0.00	0.00	0.00	0.00	0.00

The colors were highlighted based on the prevalence

(green)	0%
(yellow)	1-25%
(light orange)	26-50%
(orange)	51-75%
(red)	76-100%
